# The causal relationship between COVID-19 and increased intraocular pressure: A bidirectional two-sample Mendelian randomization study

**DOI:** 10.3389/fpubh.2023.1039290

**Published:** 2023-03-06

**Authors:** Yi Lin, Bingcai Jiang, Yuanqing Cai, Wangdu Luo, Changwei Zheng, Xiaomin Zhu, Qianyi Lin, Min Tang, Xiangji Li, Lin Xie

**Affiliations:** ^1^Department of Ophthalmology, The Third Affiliated Hospital of Chongqing Medical University, Chongqing, China; ^2^Department of Orthopaedic Surgery, The Second Affiliated Hospital of Xi'an Jiaotong University, Xi'an, Shaanxi, China

**Keywords:** COVID-19, intraocular pressure, susceptibility, severity, criticality, Mendelian randomization

## Abstract

**Background:**

Coronavirus disease 2019 (COVID-19) has brought great challenges to the global public health system and huge economic burdens to society, the causal effect of COVID-19 and intraocular pressure was blank.

**Objective:**

This study aimed to explore the causal association between coronavirus disease (COVID-19) susceptibility, severity and criticality and intraocular pressure (IOP) by bidirectional Mendelian randomization (MR) analysis.

**Materials and methods:**

Genetic associations with COVID-19 susceptibility, severity and criticality were obtained from the COVID-19 Host Genetics Initiative. Genetic associations with IOP were obtained from GWAS summary data. The standard inverse variance weighted (IVW) method was used in the primary assessment of this causality. Other methods were also implemented in supplementary analyses. Finally, sensitivity analysis was performed to evaluate the reliability and stability of the results.

**Results:**

The results showed that COVID-19 susceptibility had null effect on IOP (β = 0.131; Se = 0.211; *P* = 0.533) as assessed by the IVW method. Moreover, the results revealed that COVID-19 severity, specifically, hospitalization due to COVID-19, had a positive effect on IOP with nominal significance (β = 0.228; Se = 0.116; *P* = 0.049). However, there were null effect of COVID-19 criticality on IOP (β = 0.078; Se = 0.065; *P* = 0.227). Sensitivity analysis showed that all the results were reliable and stable. The reverse MR analysis revealed that there was null effect of IOP on COVID-19.

**Conclusions:**

We demonstrated that hospitalization due to COVID-19 might increase IOP; therefore, greater attention should be given to monitoring IOP in inpatients with COVID-19.

## 1. Introduction

Coronavirus disease 2019 (COVID-19), which is caused by severe acute respiratory syndrome coronavirus 2 (SARS-CoV-2), has brought great challenges to the global public health system and huge economic burdens to society, seriously threatening the survival of mankind (1). Presently, the pandemic is still spreading and raging in many countries globally and might last for months or years. Therefore, much attention should be given to COVID-19 with regard to etiologies, clinical manifestations, prevention and treatment, and sequelae (1–3).

COVID-19 is mainly transmitted through the respiratory tract, causing extensive upper respiratory tract infection (4). Recent reports have shown that both the cornea and conjunctiva of human eyes can express angiotensin-converting enzyme 2 (ACE2) receptor, and SARS-CoV-2 can bind to human ACE2 receptor, indicating that the eyes may be a potential entrance and storage site for SARS-CoV-2 transmission (5–7). From the point of view of the eye, conjunctivitis may be the first symptom of COVID-19; the prevalence of conjunctivitis in COVID-19 is still controversial, with estimates ranging from 0.9 to 36% (8, 9). Although uncomfortable, the symptoms of viral conjunctivitis caused by the novel coronavirus such as redness, congestion, foreign body sensation and dry eyes are not life-threatening and most cases are self-limiting; as a result, little attention has been given to the state of eye health after COVID-19 infection (10). Due to the decrease in eye care visits during the epidemic and the attention given to the more life-threatening clinical signs and symptoms of COVID-19, not much is known about the effect of COVID-19 on the eyes. Ophthalmologists still need to focus on reducing the viral load of COVID-19 in the conjunctiva and other accessory organs of the eye to prevent disease transmission whenever possible and to actively focus on ocular sequelae and intervene aggressively after COVID-19.

Intraocular pressure (IOP) is the amount of fluid pressure within the eye and is one of the most important examination metrics in ophthalmic clinics (11). It is an important marker for many ophthalmic diseases such as glaucoma and intraocular pressure that is too high or too low will damage eye tissues and visual functions to different degrees (12). Therefore, we should pay as much attention to intraocular pressure as to blood pressure. Due to the outbreak of COVID-19, people remained at home, and bad eye habits and negative emotions threatened both eye health and mental health; the situation was not conducive to intraocular pressure control (13). In particular, glaucoma is a lifelong disease, and patients need regular follow-up, resulting in a large number of glaucoma patients in the outpatient clinic; intraocular pressure is an important factor for monitoring the occurrence and development of glaucoma (14). Therefore, ophthalmologists need to pay attention to the changes in IOP after COVID-19 infection. It is worth noting that according to a previous study, there was a significant increase in IOP in patients with severe and critical COVID-19 disease (15). Therefore, it is reasonable to think that in the autoimmune stage of COVID-19, a considerable number of patients may exhibit subclinical ocular inflammation and possibly high intraocular pressure (11). If high IOP is not dealt with in time, it may lead to irreversible vision loss. However, it is still unknown whether IOP increases after COVID-19 infection and this question require further study.

**Figure 1:**
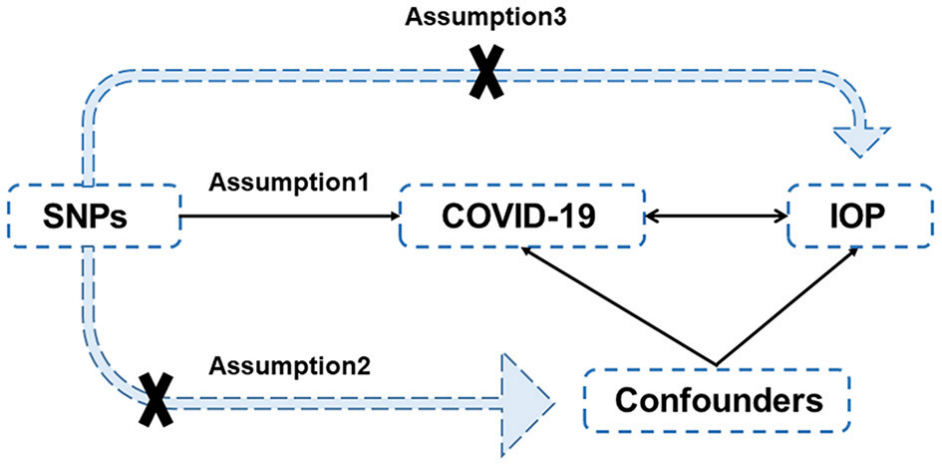
Diagram of Mendelian randomization. There are three assumptions: (1) the IVs, (SNPs in this case) are closely associated with the exposures; (2) the IVs are independent of confounders of exposures and outcomes; (3) the IVs affect the outcomes only through the exposures and not by direct action. SNP, single-nucleotide polymorphisms; COVID-19, coronavirus disease 2019; IOP, intraocular pressure.

Mendelian randomization has been widely used in etiology research in recent years. In Mendelian randomization (MR) research, exposure is regarded as an intermediate phenotype, which is determined by genotype (16). Genotype variants [generally single nucleotide polymorphisms (SNPs)] are used as instrumental variables (IVs) to study the causal relationship between genotypes and diseases to simulate the relationship between exposure and diseases. Therefore, MR analyses are not affected by the bias and confounders that affect existing traditional epidemiological methods (such as retrospective research), and reverse causality can also be avoided. Conceptually, it is similar to prospective randomized controlled trials (RCTs), although MR can be performed retrospectively (Figure 1). Since all hereditary genetic variations are determined at conception and occur before diseases, MR can avoid non-differential measurement errors or mixed potential deviations. Since it is difficult to collect clinical data, we use MR to assess the causal relationship between COVID-19 and IOP to provide indirect evidence for clinical practice. In this study, we aim to explain the observational relationship between COVID-19 and intraocular pressure and predict the risk of ocular hypertension caused by COVID-19 in the future by using a two-sample MR analysis.

## 2. Methods

### 2.1. Study design

In the present study, a two-sample bidirectional MR study was performed to assess the causal association between COVID-19 and IOP. Single-nucleotide polymorphisms (SNPs) were selected as instrumental variables (IVs). Generally, three assumptions should be met for IVs (Figure 1): (1) the IVs (in this case, SNPs) are highly associated with the exposures; (2) the IVs are independent of confounders of exposures and outcomes; and (3) the IVs only impact the outcomes through the exposures, rather than by direct effect.

### 2.2. Data sources

The summary statistics of COVID-19 susceptibility, COVID-19 severity and COVID-19 criticality were obtained from the COVID-19 Host Genetics Initiative [COVID-19 Host Genetics (17)], which was based on subjects of European ancestry and included genome-wide association studies (GWAS) summary data with regard to different COVID-19 outcomes. Susceptibility was defined as testing positive for COVID-19, severity was defined as hospitalization with COVID-19, and criticality was defined as very severe respiratory symptoms due to COVID-19 as compared to population controls. In the 5th release, there were 38,984 COVID-19 cases (with 1,644,784 controls), 9,373 cases hospitalized due to COVID-19 (with 1,197,256) and 4,792 cases suffering from very severe respiratory symptoms due to COVID-19 (with 1,054,664 controls). Age and gender composition were not provided in these data. The summary statistics of IOP were obtained from GWAS meta-analyses summary data, which was published by Springelkamp et al. (18), a meta-analysis of GWAS assessing IOP in the European cohort was conducted with a sample size of 29,578 individuals and the findings were validated in multiple sets of POAG cases and controls.

### 2.3. Instrumental variables extraction

For MR analysis, those strongly associated with COVID-19 susceptibility, severity and criticality or IOP SNPs (*P* < 5^*^10^−8^) with minor allele frequency (MAF) >0.01 were extracted, and SNPs related to confounders assessed by Phenoscanner (http://www.phenoscanner.medschl.cam.ac.uk/) were removed. Next, a clumping process was performed (*r*^2^ < 0.01, window size = 5,000 kb) to ensure that all the SNPs chosen for the MR analysis were not in linkage disequilibrium (LD) since bias could be introduced when instrumental SNPs are in strong LD. Then, harmonizing was conducted to ensure that the effect allele of IVs in exposure and outcome was consistent across different databases. Moreover, to avoid biases caused by weak IVs, *F* statistics (*F* statistics = β^2^/SE^2^) were calculated for each SNP, and SNPs whose *F* statistics were <10 were considered to be weak IVs and excluded. In addition, a Steiger filtering test was performed to remove IVs that explained more of the variance in the outcome than in the exposure, ensuring the absence of reverse causality in MR analysis. Finally, the MR-PRESSO test was performed to detect potential outliers and the outliers were removed after being identified. The MR-PRESSO outlier test requires that at least 50% of the variants are valid instruments, has balanced pleiotropy, and relies on the Instrument Strength Independent of Direct Effect (InSIDE) condition that instrument-exposure and pleiotropic effects are uncorrelated (19, 20).

### 2.4. Statistical analysis

In this study, the inverse variance weighted (IVW) method was primarily used to evaluate the causal relationship between COVID-19 and IOP. The IVW method is a method of aggregating and minimizing the sum of the variances of two or more random variables that involves a weighted linear regression model in which each random variable is assigned a weight inversely proportional to its variance. To assess this causality comprehensively, other MR methods, including MR-Egger, weighted median, simple mode, and weighted mode, MR-PRESSO were also applied. MR-Egger regression is similar to the IVW method and also uses data on the genetic variation in disease and exposure factors. In traditional IVW, the intercept in the linear regression model is forced to be zero, whereas in MR-Egger regression, an intercept is estimated in the regression equation and a statistically significant non-zero intercept term indicates the presence of directional bias in the selected genetic variants. Finally, reverse MR analysis was performed.

Cochran's *Q*-test was used to test for heterogeneity and a potential pleiotropy test was implemented using the MR-Egger intercepts and MR-PRESSO. All statistical analyses were performed in R software (Version 3.6.1) using the R 153 package “Two sample MR” (version 0.5.6). Bonferroni correction was used to adjusted the *P*-value, A *P-*value < 0.0167(0.05/3) was considered to be statistical significant.

## 3. Results

### 3.1. The extraction of instrumental variables

In the present work, the SNPs strongly related to the exposures were chosen as IVs for MR analysis. Ultimately, there were 4 COVID-19 susceptibility-related SNPs with a mean *F*-statistic of 68.20: rs10936744, rs2271616, rs35508621, rs4971066. There were 6 (rs10774671, rs111837807, rs1819040, rs1886814, rs35081325, and rs72711165), and 7 (rs10735079, rs10860891, rs111837807, rs13050728, rs2109069, rs622568, and rs77534576) related to hospitalizations and very severe respiratory symptoms due to COVID-19, with means of 73.55 and 49.42, respectively. And there were 9 IOP-related SNPs with a mean *F*-statistic of 41.23: rs10281637, rs10892569, rs10918274, rs11039603, rs12912010, rs149758779, rs2472493, rs6445055, and rs9913911. The MR-PRESSO test showed that there were no outliers. The details of the SNPs are available in Supplementary material 1–4.

### 3.2. The causal association between COVID-19 and IOP

**Figure 2:**
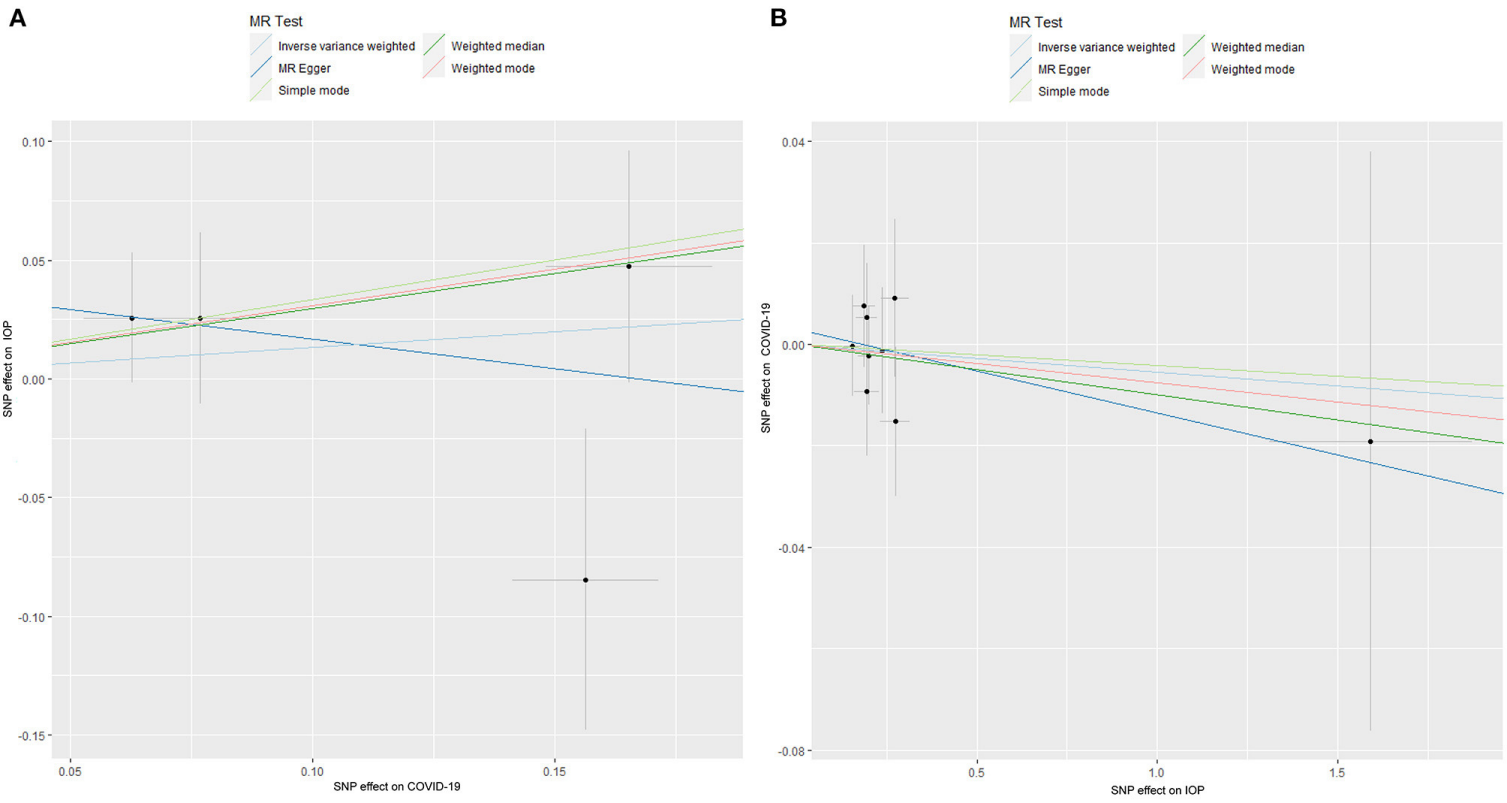
The Mendelian randomization analysis result of COVID-19 susceptibility and IOP **(A)**, and reverse MR analysis **(B)**. IOP, intraocular pressure; COVID-19, coronavirus disease; SNP, single nucleotide polymorphism; MR, Mendelian randomization.

The results of the MR analysis of the causal effect of COVID-19 susceptibility on IOP are summarized in Table 1 and Figure 2A. As shown in Figure 2A, COVID-19 susceptibility had null causal effect on the IOP (β = 0.131; Se = 0.211; *P* = 0.533) estimated by the IVW method. The other MR methods, including MR Egger (β = −0.249; Se = 0.555; *P* = 0.698), Weighted median (β = 0.296; Se = 0.241; *P* = 0.219), Weighted mode (β = 0.308; Se = 0.289; *P* = 0.366) also confirmed that there was no effect of COVID-19 susceptibility on IOP. The reverse MR analysis results were summarized in Table 2 and Figure 2B. The results showed that there were no genetic causal association between IOP and COVID-19 susceptibility analyzed by IVW [odds ratio (OR): 0.995; 95%CI: 0.961–1.029; *P* = 0.757], the results were further verified by MR Egger (OR: 0.984; 95%CI: 0.912–1.061; *P* = 0.683), Weighted median (OR: 0.990; 95%CI: 0.948–1.034; *P* = 0.653), Simple mode (OR: 0.996; 95%CI: 0.937–1.059; *P* = 0.896), Weighted mode (OR: 0.992; 95%CI: 0.933–1.056; *P* = 0.818).

**Table 1:** Mendelian randomization (MR) analysis results with regards to causal effect of COVID-19 on IOP.

**Exposures**	**Method**	**SNP (*n*)**	**β**	**Se**	***P*-value**
COVID-19 susceptibility	MR egger	4	−0.249	0.555	0.698
	Weighted median	4	0.296	0.241	0.219
	Inverse variance weighted	4	0.131	0.211	0.533
	Simple mode	4	0.334	0.327	0.382
	Weighted mode	4	0.308	0.289	0.366
Hospitalization due to COVID-19	MR Egger	6	0.518	0.515	0.371
	Weighted median	6	0.265	0.145	0.068
	Inverse variance weighted	6	0.228	0.116	0.049
	Simple mode	6	0.364	0.214	0.149
	Weighted mode	6	0.319	0.192	0.159
Very severe respiratory symptoms due to COVID-19	MR egger	7	0.004	0.474	0.993
	Weighted median	7	0.086	0.081	0.291
	Inverse variance weighted	7	0.078	0.065	0.227
	Simple mode	7	0.232	0.131	0.128
	Weighted mode	7	0.154	0.118	0.241

COVID-19, coronavirus disease; IOP, intraocular pressure; SNP, single nucleotide polymorphism.

**Table 2:** Mendelian randomization (MR) analysis results with regards to causal effect of IOP on COVID-19.

**Outcomes**	**Method**	**OR**	**95%CI**	***P*-value**
COVID-19 susceptibility	MR egger	0.984	0.912–1.061	0.683
	Weighted median	0.990	0.948–1.034	0.653
	Inverse variance weighted	0.995	0.961–1.029	0.757
	Simple mode	0.996	0.937–1.059	0.896
	Weighted mode	0.992	0.933–1.056	0.818
Hospitalization due to COVID-19	MR egger	1.002	0.673–1.494	0.991
	Weighted median	0.985	0.903–1.076	0.740
	Inverse variance weighted	0.975	0.911–1.043	0.458
	Simple mode	0.990	0.867–1.131	0.891
	Weighted mode	0.992	0.867–1.134	0.905
Very severe respiratory symptoms due to COVID-19	MR egger	0.945	0.474–1.883	0.877
	Weighted median	0.934	0.807–1.082	0.365
	Inverse variance weighted	0.928	0.829–1.039	0.193
	Simple mode	0.991	0.771–1.274	0.947
	Weighted mode	0.969	0.759–1.237	0.809

COVID-19, coronavirus disease; IOP, intraocular pressure; OR, odds ratio; SNP, single nucleotide polymorphism; CI, confidence interval.

### 3.3. The causal relationship between COVID-19 severity and IOP

**Figure 3:**
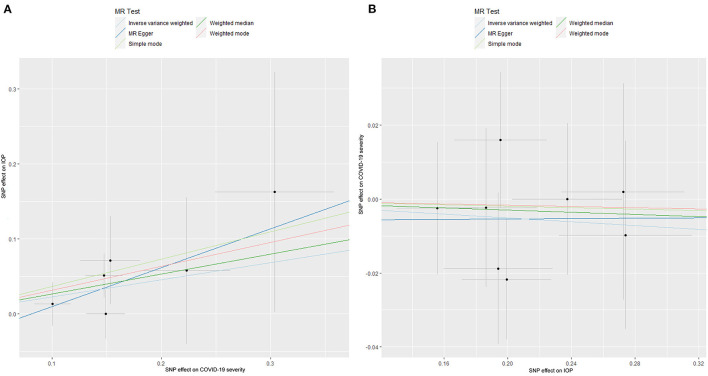
The Mendelian randomization analysis result of COVID-19 severity and IOP **(A)**, and reverse MR analysis **(B)**. IOP, intraocular pressure; COVID-19, coronavirus disease; SNP, single nucleotide polymorphism; MR, Mendelian randomization.

**Figure 4:**
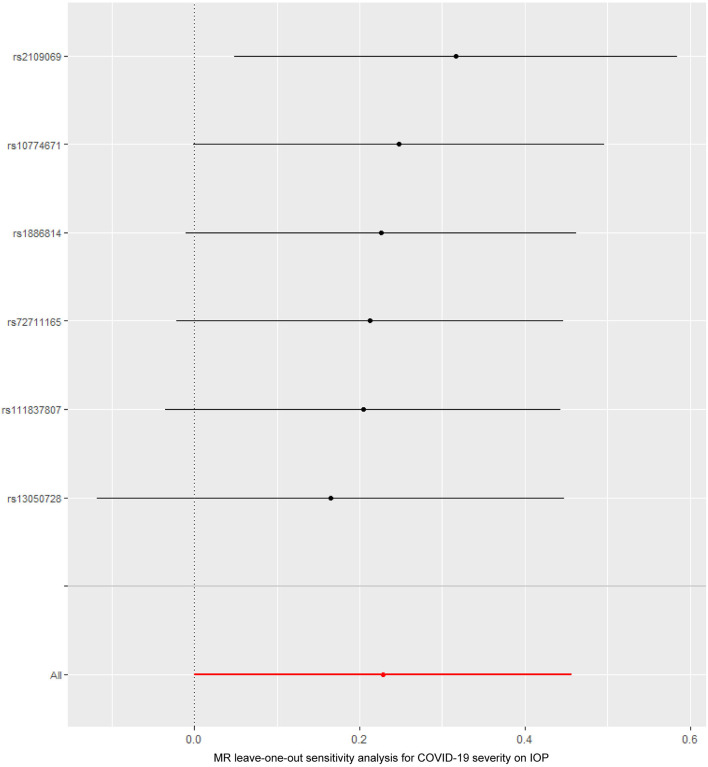
The Mendelian randomization leave-one-out sensitivity analysis for COVID-19 severity on IOP. IOP, intraocular pressure; COVID-19, coronavirus disease; MR, Mendelian randomization.

The causal relationship between COVID-19 severity, namely, hospitalization due to COVID-19, and IOP assessed by MR is summarized in Tables 1, 2 and Figure 3. The results showed that hospitalization due to COVID-19 had a positive effect of IOP (β = 0.228; Se = 0.116) with nominal significance (*P* = 0.049) (Figure 3A) analyzed by IVW, indicating that hospitalization due to COVID-19 might increase IOP. Cochran's *Q*-test showed that the absence of heterogeneity (*Q*-value = 2.276; *P* = 0.809) of MR analysis results, and MR-Egger intercept test (MR-Egger intercept = −0.0419; Se = 0.0725; *P* = 0.595) and MR-PRESSO test both showed no evidence of pleiotropy, the stability and reliability of MR analysis results were verified by the leave-one-out test (Figure 4). The reverse MR analysis results were summarized in Table 2. As the table showed, there was null effect of IOP on COVID-19 severity assessed by IVW (OR: 0.975; 95% CI: 0.911–1.043; *P* = 0.458), and other MR methods, including MR Egger (OR: 1.002; 95% CI: 0.673–1.494; *P* = 0.991), Weighted median (OR: 0.985; 95% CI: 0.903–1.076; *P* = 0.740), Simple mode (OR: 0.990; 95% CI: 0.867–1.131; *P* = 0.891), Weighted mode (OR: 0.992; 95% CI: 0.867–1.134; *P* = 0.905) (Figure 3B), also confirmed the null causal effect of IOP on COVID-19 severity.

### 3.4. The causal COVID-19 criticality on IOP

**Figure 5:**
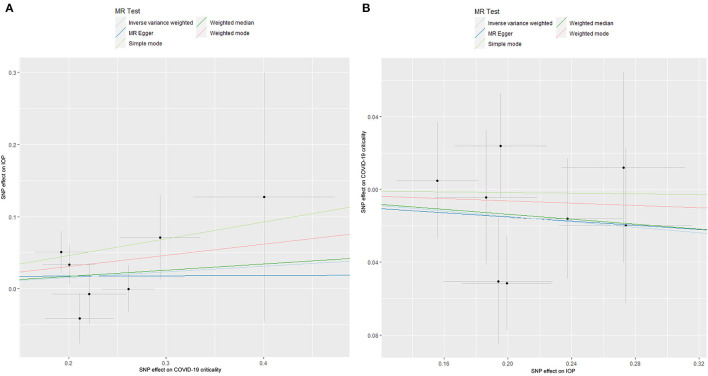
The Mendelian randomization analysis result of COVID-19 criticality and IOP **(A)**, and reverse MR analysis **(B)**. IOP, intraocular pressure; COVID-19, coronavirus disease; SNP, single nucleotide polymorphism; MR, Mendelian randomization.

The causal association between COVID-19 criticality, defined as very severe respiratory symptoms confirmed to be due to COVID-19 infection, and IOP is summarized in Tables 1, 2 and Figure 5. As shown by the results of the IVW analysis, very severe respiratory symptoms confirmed to be due to COVID-19 infection have null effect on IOP (β = 0.078; Se = 0.065; *P* = 0.227), which was further verified by other methods, such as the MR Egger (β = 0.004; Se = 0.474; *P* = 0.993), Weighted median (β = 0.086; Se = 0.081; *P* = 0.291), Weighted mode (β = 0.154; Se = 0.118; *P* = 0.241) (Table 1, Figure 5A). Moreover, the reverse MR analysis were also performed, and the results were summarized in Table 2 and Figure 5B. The results showed that there was null effect of IOP on COVID-19 criticality estimated by IVW (OR: 0.928; 95% CI: 0.829–1.039; *P* = 0.193), and these results were further verified by MR Egger (OR: 0.945; 95% CI: 0.474–1.883; *P* = 0.877), Weighted median (OR: 0.934; 95% CI: 0.807–1.082; *P* = 0.365), Simple mode (OR: 0.991; 95% CI: 0.771–1.274; *P* = 0.947), Weighted mode (OR: 0.969; 95% CI: 0.759–1.237; *P* = 0.809).

## 4. Discussion

Recently, there more and more MR analysis with regards to COVID-19, for example, Sun et al. (21) performed a large-scale two-sample MR analysis to assess the causal roles of many traits in severe COVID-19, which would be helpful for guiding the effective protection of high-risk individuals. In this study, large-scale GWAS summary data were used to explore the causal relationship between COVID-19 and IOP with two-sample MR for the first time. The results showed that there was a positive causal relationship between COVID-19 severity and IOP with nominal significance.

COVID-19, first reported in Wuhan, China, has developed into a devastating worldwide pandemic that has posed severe challenges to public health (8). There are many complications of COVID-19, including cardiovascular adverse events (such as myocarditis, stroke, etc.), pulmonary fibrosis after COVID-19 infection and others (22–24). In addition, the eye surface has attracted the interest of researchers as a possible transmission medium; it may be a direct contact site providing another channel for viruses to enter the respiratory system, or the virus might spread through infected conjunctiva or tears (25). Regarding ocular implications, it has been reported that COVID-19 infection results in conjunctivitis, diabetic retina, and neurological complications, including polyneuritis (26); for patients treated in intensive care units, the main complications are ocular surface disorders, ranging from mild conjunctival irritation to severe infectious keratitis, and rare ocular complications, including acute ischemic optic neuropathy, resulting in permanent vision loss (26). Therefore, COVID-19 infection may cause different degrees of damage to eye tissues. IOP is an indispensable index in clinical practice and an important reference for doctors to assess eye health. Presently, the impact of COVID-19 infection on IOP is still unclear. Szkodny et al. (27) performed a cross-sectional study and demonstrated that compared with controls, there were no significant differences in IOP in patients with COVID-19. Another case-control study performed by Savastano et al. (28) also showed the same result. However, these conclusions might be unreliable due to potential biases and confounders in these studies, since most of these studies were cross-sectional or retrospective studies and the sample sizes were small. Costa et al. (15) conducted a study with 64 COVID-19 patients who were classified as mild-to-moderate, severe, and critical and concluded that the IOP in critical cases (14.16 ± 1.88 mmHg) was significantly higher than that in severe cases (12.51 ± 2.40 mmHg), both in the right (*P* = 0.02) and left eyes (*P* = 0.038), indicating that COVID-19 infection might be related to higher IOP, although only nominal significant replication in the present study. However, it is worth noting that this was a study with small sample size and there was apparent selection bias regarding patient recruitment, leading to inconclusive results. Thus, more research is warranted to reach a more precise conclusion.

As a novel strategy for research in causality, MR has appealed to many investigators in recent years. It has been widely applied in diverse studies. MR can avoid the influence of confounding factors and indirectly infer the causal relationship between exposure and outcome using genetic variation related to exposure factors, which is more practical and convenient than the gold standard randomized controlled trial (RCT) to verify the causal relationship and similar to RCT in terms of reliability. In this study, SNPs closely related to exposure were extracted from large samples of GWAS data based on subjects with European ancestry, and causality analysis was carried out by two-sample MR. Causality was evaluated by various MR methods, such as the IVW, MR–Egger regression and weighted median estimation, and sensitivity analyses were conducted to test for potential heterogeneity and pleiotropy, which proved the stability and reliability of the MR results. In this study, we first applied MR, an emerging tool for epidemiological studies, to estimate the causal relationship between different COVID-19 outcomes and IOP, and we showed that some of the results were similar to those of Costa et al. (15). COVID-19 severity a exhibited a positive effect on IOP, which were analyzed by the IVW method, and the sensitivity analysis showed that the results were reliable and stable. Although it is known that there is a clear relationship between many kinds of anterior chamber virus infections, including cytomegalovirus and herpes simplex virus-1, and intraocular pressure elevation, the mechanism is still unclear. At the genetic level, this study also verified that there is an obvious correlation between COVID-19 infection and IOP. The potential mechanism might be mediated by a cytokine storm, also known as cytokine release syndrome (CRS), caused by COVID-19 infection, which affects the imbalance of aqueous humor production and outflow (29). Another explanation might also be discussed from the nerve perspective. COVID-19 is considered a potential neurotrophic virus that is also toxic to nerves. It is possible to cause a central intraocular pressure increase by infecting intraocular pressure into the nerve pathway and interfering with the nucleus related to contralateral hypothalamus intraocular pressure regulation (30). The specific mechanisms warrant further exploration.

Our research results show that severe COVID-19 might increase the risk of ocular hypertension, so we suggest that patients with COVID-19, especially those with severe COVID-19, be watchful for ocular hypertension to prevent its occurrence. During the outbreak of COVID-19, telemedicine gradually entered the mainstream and greatly changed the way patients sought medical treatment. Many patients were reluctant to go to the hospital for fear of being infected. In addition, during the epidemic, medical resources were routed to departments in urgent need, such as respiratory and critical illness departments, which reduced the number of outpatient clinics in ophthalmology. At the same time, the transmission mode of COVID-19 was highly correlated with ophthalmology, which made people panic about going to the ophthalmology department. Recently, it has been reported in the literature that during the COVID-19 pandemic, the compliance of glaucoma patients with intraocular pressure-lowering drugs worsened, which seems to be related to patients' adaptability, and one effect of the pandemic may be increased vision problems. Therefore, for COVID-19 patients, especially those suffering from glaucoma, it is strongly recommended to increase the frequency of intraocular pressure assessments. In addition, timely public education regarding the avoidance of unhealthy lifestyles and targeted guidance for high-risk glaucoma patients during the epidemic may effectively reduce the acute angle-closure glaucoma and reduce the medical burden (31).

There were some limitations of this study: (1) this two-sample MR analysis was based on individuals of European ancestry and whether this causality applies to other ancestries is unclear; (2) the specific molecular mechanism for this relationship has still not been determined and more basic research should be performed to elucidate the pathophysiological process; and (3) it is unclear that whether increased IOP is present in more severe COVID-19 infections, more work should be performed to address this issue.

In summary, this study first investigated the causal effect of COVID-19 on IOP by two-sample MR and the results showed that severe COVID-19 exhibited a positive effect on IOP, indicating that much attention should be given to IOP monitoring in COVID-19 patients.

## Data availability statement

Publicly available datasets were analyzed in this study. This data can be found here: https://gwas.mrcieu.ac.uk/.

## Author contributions

YL, YC, and WL performed the study and wrote the manuscript. CZ, XZ, and BJ collected data. QL, MT, and XL interpret the results. LX designed this study. All authors contributed to the article and approved the submitted version.

## Supplementary material

Supplementary material 1 The details about single nucleotide polymorphisms of COVID-19 susceptibility selected for Mendelian randomization analysis.

Supplementary material 2 The details about single nucleotide polymorphisms of COVID-19 severity selected for Mendelian randomization analysis.

Supplementary material 3 The details about single nucleotide polymorphisms of COVID-19 criticality selected for Mendelian randomization analysis.

Supplementary material 4 The details about single nucleotide polymorphisms of intraocular pressure selected for Mendelian randomization analysis.
